# A functional learning health system in Japan: Experience with processes and information infrastructure toward continuous health improvement

**DOI:** 10.1002/lrh2.10252

**Published:** 2020-11-12

**Authors:** Hidehisa Soejima, Kautarou Matsumoto, Naoki Nakashima, Yasunobu Nohara, Takanori Yamashita, Jiro Machida, Hideki Nakaguma

**Affiliations:** ^1^ Saiseikai Kumamoto Hospital Kumamoto Japan; ^2^ Faculty of Advanced Science and Technology Kumamoto University Kumamoto Japan; ^3^ Medical Information Center Kyusyu University Hospital Fukuoka Japan; ^4^ Urology Saiseikai Kumamoto Hospital Kumamoto Japan

**Keywords:** improvement, pathway, variance

## Abstract

Introduction and definition of the term Learning Health System (LHS) appears to have occurred initially around 2007. Prior to this and the introduction of electronic health records (EHR), a predecessor could be found in the Clinical Pathways concept as a standard medical care plan and a tool to improve medical quality. Since 1997, Japan's Saiseikai Kumamoto Hospital (SKH) has been studying and implementing Clinical Pathways. In 2010, they implemented EHR, which facilitated the collection of structured data in common templates that aligned with outcome measurements defined through Japan's Society of Clinical Pathways. For each patient at this hospital, variances from the desired outcomes have been recorded, producing volumes of structured data in formats that could readily be aggregated and analyzed. A visualization tool was introduced to display graphs on the home page of the EHR such that each patient can be compared to similar patients. Knowledge learned from patient care is shared regularly through Clinical Pathways meetings that are supported by all staff within the hospital. The SKH experience over the past two decades is worth exploring further in the context of the development of a fully functional LHS and the attributes/characteristics thereof. In this report, the SKH experience and processes are compared with previously published attributes of a fully functional LHS (ie, characteristics of an LHS that can indicate maturity). Specific examples of the SKH system are detailed with respect to leveraging knowledge gained to change performance that improves patient care as prescribed by learning health cycles. The SKH experience and its information infrastructure and culture exemplify a functional LHS, which is now being expanded to additional hospitals with the hope that it can be scaled and serve as a solid platform for measures aimed at improving medical care, thus establishing broader and more global learning health systems.

## 1 INTRODUCTION

A “learning health system” (LHS) has been defined by the U.S. National Academies of Sciences, Engineering and Medicine (formerly the Institute of Medicine) as “…one in which science, informatics, incentives and culture are aligned for continuous improvement and innovation, with best practices seamlessly embedded in the delivery process and new knowledge captured as an integral by‐product of the delivery experience.”^1^ Additional progress to better define and further LHS progress was made during an LHS Summit convened by the Joseph H. Kanter Family Foundation (KFF) in 2012 where a set of LHS Core Values was established and the Learning Health Community (LHC) was formed.^2^ The LHC has posted the LHS Core Values, organized meetings to develop a Consensus Action Plan,^3^ and more recently incorporated as a 501c3 nonprofit organization. Key initiatives of the LHC have been to sponsor a Bridging Collaborative Conference in 2019,^4^ which explored barriers and stimuli toward bridging research and health care to support LHSs, and to build consensus around an LHS Maturity Model.

In 2017, Friedman, Rubin, and Sullivan^5^ published a vision of LHSs, connecting this vision to the learning health cycle (the fundamental process of an LHS) and emphasizing the importance of information infrastructure and culture. They describe additional coordination and initiatives that would ideally evolve toward an information infrastructure and a culture that will support the improvement of global health. Specifically, they describe five characteristics or attributes of a fully functioning LHS that would adhere to the LHS Consensus Core Values, complete learning health cycles, and leverage robust infrastructure and corporate culture. Adherence to these five characteristics/attributes of a fully functional LHS may be a step in the direction of building consensus toward a global LHS maturity model.

Around the world, hospitals and academic health organizations are striving to develop robust LHSs. Barriers to reaching this vision are many, including lack of robust information, insufficient dissemination of learnings, and the varied implementations and data models used by electronic health record (EHR) vendors and researchers. Although, over time, the goal will be a global LHS, the most feasible LHS is currently one that is developed within the confines of an individual institution or to support a specific network. Thus, the Saiseikai Kumamoto Hospital (SKH) experience over the past two decades is worth exploring further in the context of the development of a fully functional LHS and the attributes/characteristics thereof.

The eventual goal is for institution‐based LHSs to share knowledge across multiple institutions and more broadly to support global health. This may require concentric learning health cycles initially; however, access to large volumes of data in comprehensive formats and broad adoption of standards to enable interoperability will facilitate sharing of knowledge and information between the cycles and among institutions or networks.

## 2 BACKGROUND

Introduction and definition of the term Learning Health System (LHS) appears to have occurred initially around 2007.^1^ Prior to that time, and prior to the introduction of electronic medical records (EMR) or EHR, a related concept of standardizing and continuously improving processes for patient care had been evolving; the genesis of these care protocols, called “Clinical Pathways” or “Integrated Care Pathways,” seems to be attributed to Zander et al who authored a book published in 1985, entitled “Nursing Case Management, Blueprints for Transformation” and thereafter published a number of related articles^6^, ^7^ There are now Clinical Pathways Societies around the world. There have been varied definitions over the years^8^ but the Japanese Society for Clinical Pathways (JSCP) has defined a Clinical Pathway (CP) as a standard medical care plan that includes patient condition and medical treatment and evaluation goals, recording, and a tool to improve medical quality by analyzing deviation from the standard.^9^

SKH is a member of the Saiseikai Group, located in Kumamoto City in the center of Kyushu, Japan. It is Joint Commission International (JCI) accredited as an acute care hospital with 400 beds and a critical care center with a total of 2044 employees. For more than two decades, Japan's SKH has been studying and implementing Clinical Pathways. Various forms of Clinical Pathways were developed in many hospitals; however, they were initially paper‐based, and the importance of standardization of data collection to support these was not emphasized. Clinical Pathways were introduced into the SKH in Japan in 1996 to improve medical quality. The approach was one of continuous improvement (*kaizen* in Japan), and it was also initially a paper‐based process. At SKH, there have been regular (every other month) hospital‐wide meetings called Clinical Pathways Conferences, which are led by various hospital teams/departments since 1997 to discuss the Clinical Pathways (care plans) and how to improve them.

Applying the *kaizen* principles along with integrating new technology to obtain structured digital health information enabled the development of a hospital‐wide system that has now been shown to significantly improve the functioning of the hospital, producing better patient outcomes at lower costs. The methodology and technology supporting the electronic collection of large volumes of high‐quality data were initiated in 2010 when EHR were implemented at SKH. Views of the data and graphic depictions of ongoing data collection are now readily displayed for all hospital staff to view at any time, and machine learning and artificial intelligence are now being applied to learn from the data. SKH received the Gold Seal of approval from the JCI^10^ in 2013 and has maintained this rating each year since that award.

Based upon the numerous methods and requirements that were developed and/or met in order to achieve the current status, the SKH experience appears now to represent a functional learning health system. More recently, there has been keen interest by Japan's Agency for Medical Research and Development (AMED)^11^ to fund the extension of this approach and technology to extract real‐world medical data beyond hospitals and vendors. This project is reinforcing the importance of consensus‐based standards and definitions for the collection of sufficient high‐quality data that lead to quality measurements to support continuous improvement and learning for improving health and reducing costs. This expansion is also adding concentric simultaneous learning health cycles to the one within SKH.

Each Clinical Pathways Conference at SKH presents knowledge derived from the collection of data from all patients who visit the hospital. Interested parties from other areas of Japan and other countries attend these meetings and contribute relevant comments and information. In turn, they take this information back to their own hospitals. One collaborating hospital is Kyushu University. In this report, the experiences from SKH and Kyushu University Hospital are described in the context of published attributes of fully functional LHS, which could potentially support an LHS maturity model.

### 2.1 Characteristics and attributes of a fully functional LHS

The characteristics/attributes described by Friedman et al for a fully functional LHS are paraphrased and abbreviated below. These attributes were described to serve a number of different purposes, including measuring progress, identifying success factors, enabling LHSs to learn from one another, and potentially to inform a future LHS maturity model.^5^ The first three attributes are aligned with the concept of a learning health cycle, with data being converted to knowledge (D2K), knowledge influencing performance (K2P), and changes in performance generating further data (P2D).^12^ The SKH “Electronic Clinical Pathways” approach and related experiences over the past three decades have been compared to these LHS attributes as a “test” of LHS maturity. The details and related progress are described in the following section.

1. **Data to Knowledge**: Health‐related characteristics, experiences, and other relevant types of data are available securely from a large population of individuals. This is the Data to Knowledge (D2K) component of a learning cycle, which requires that data be available in sufficient quantity with sufficient quality to generate findings that are credible. Routine collection and protected access and storage of such data are important.
2. **Knowledge to Performance**: Health‐related decisions and actions by stakeholders are supported with best practice knowledge derived from the D2K data; this is referred to as the Knowledge to Practice (K2P) component of a learning cycle. Robust K2P can support recommendations to all health ecosystem participants at various levels, from the individual to the system level.
3. **Continuous Learning and Health Improvement (Performance to New Data)**: Health improvement and learning are continuous and routine. The recognition that such continuous improvement is essential and the best way to decrease costs and provide high quality, safe health care, even in the absence of imposing external stimuli or crises. This completes the cycle from performance to the generation of new data (P2D). Multiple learning cycles may occur simultaneously.
4. **Shared Infrastructure**: Multiple learning cycles are routine and enabled by shared infrastructure, including technologies, policies, supportive services, and standards.
5. **Culture**: Within the LHS, stakeholders see the value and view the related activities as integral to their culture. Learning cycles will be supported by and motivated through learning communities comprised of diverse groups of individuals who are driven to removing barriers, reducing costs, and improving safety while ultimately improving health.

### 2.2 Comparison of Saiseikai Kumamoto System with attributes of a fully functional LHS

#### 2.2.1 Data to Knowledge (D2K)

Within the SKH, data have been gathered on every patient since the initiation of the idea of Clinical Pathways in 1996. The data collection was paper‐based at that time. However, in 2010, an EHR system was introduced, making it possible to collect and share patient data electronically. The lack of standardization of the data entered, however, made it impossible to analyze across patients for the purpose of learning from each patient.

In 2011, a set of consensus‐based standards for this purpose was developed, working with the JSCP. SKH began to implement these initial formats for data collection. The concept involved developing a way to record assessments that reflect defined and acceptable outcomes or detect if there were “variances” from the acceptable range. The latter would indicate that a patient's condition or course of therapy was deviating from the acceptable range. Data were collected in a controlled and structured manner through templates such that these comparisons and analyses could be accomplished.

Specifically, SKH working with JSCP developed a set of “Basic Outcome Master” (BOM) templates for collecting the data relevant to specific care protocols and sets of Outcome‐Assessment‐Task (OAT) Units that define the assessments that support each outcome along with the personnel tasks that are required to take these assessments. The OAT unit is considered the basic or minimum unit of medical care.

**FIGURE 1:**
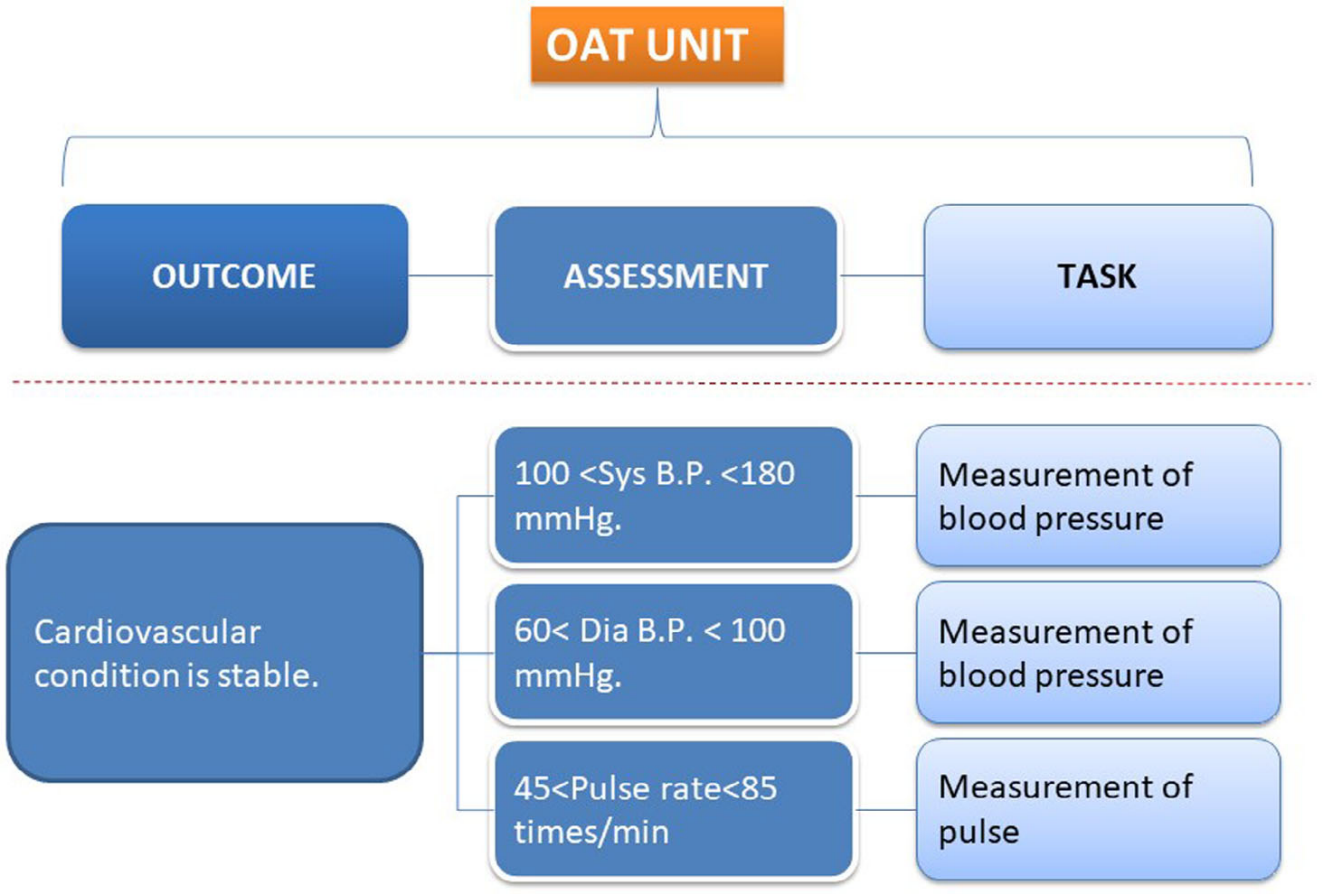
OAT Unit as the Basic Unit of Medical Care: Outcome, Assessment, and Task are related to each other. The OAT unit is available for data modeling. An outcome of a clinical pathway is our clinical goal and its achievement is judged by assessments with tasks. If our expected outcome is not obtained, the situation is called a variance

For example, one BOM could be “Discharge Safely from the Hospital After Surgery.” The measures that would determine this outcome would be related to a stable condition after surgery, specifically: stable cardiovascular condition, defined to meet certain criteria (eg, systolic blood pressure between 100 and 180 mm Hg, diastolic blood pressure between 60 and 100 mm Hg, pulse rate of 45‐85 beats/min). The measures would include blood pressure and pulse. “Assessments” involve a “Task” such as “measurement of blood pressure”. Each OAT unit has one outcome and several assessments with associated tasks (Figure 1).

**FIGURE 2:**
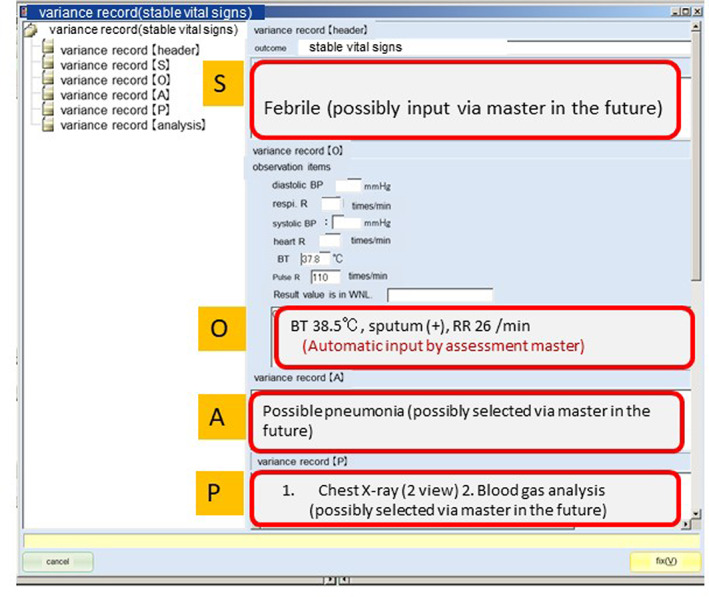
SOAP recording using a dynamic template (S, Subjective; O, Objective; A, Assessment; P, Plan) Variances from what is considered normal are recorded in the template. Templates for each outcome are provided. Input is in the S, O, A, P free format in relation to variance. If items of assessment and plan are in the form of structured master templates, it is easy to collect higher‐quality structured data for analysis and to inspect the validity of assessment and treatment

**FIGURE 3:**
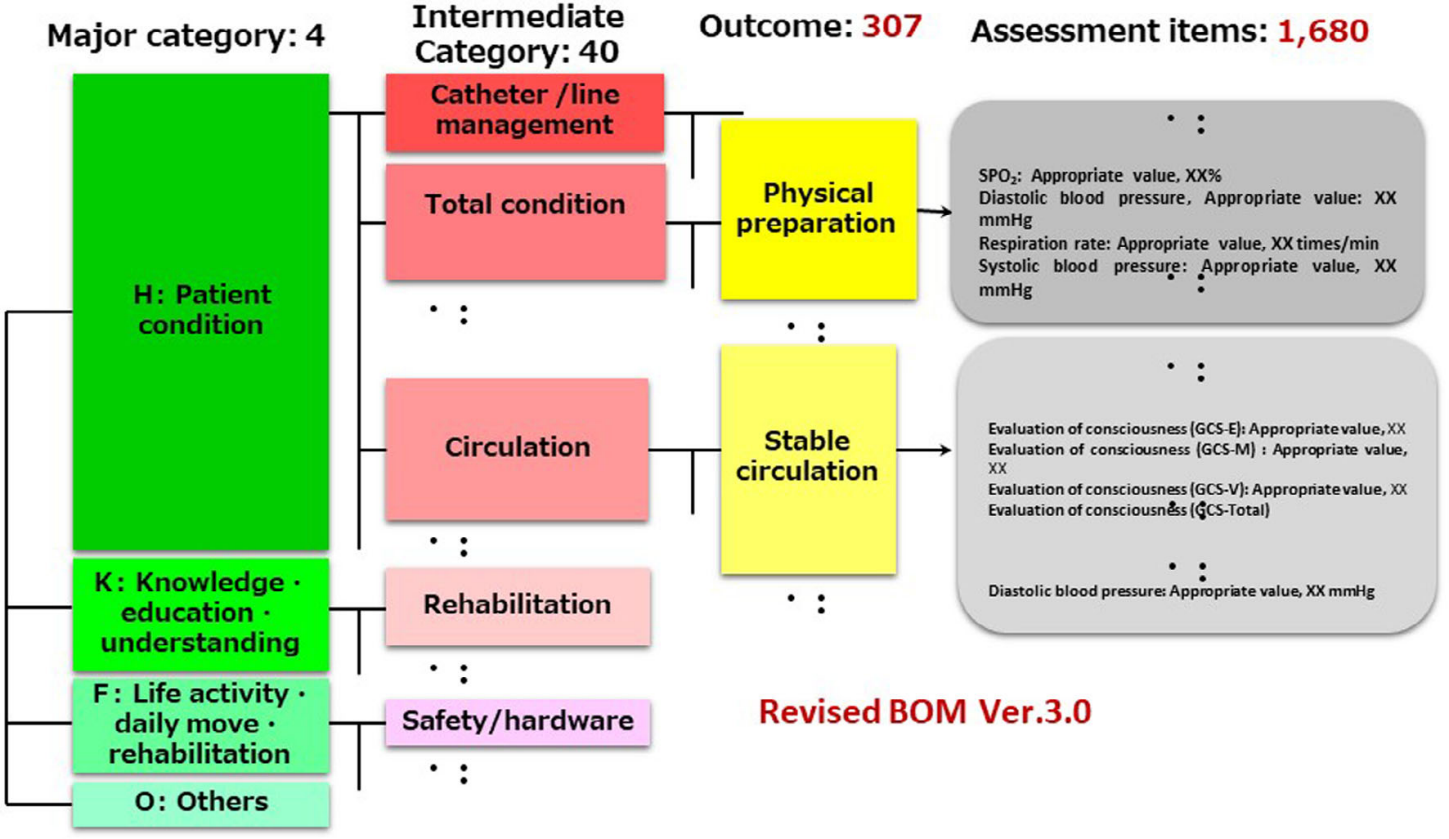
Structure of BOM (Basic Outcome Master) by Japanese Society for Clinical Pathway (JSCP) BOM has four layers, with the most important layer being outcome. An outcome and several related assessments are selected. Narrative medical sentences can be expressed by BOM, which is collectable as structured data

SKH staff have been completing interactive BOM templates and related assessments for every patient for over a decade now, thus collecting relevant data to enable an understanding of the experiences of each patient who comes to this healthcare institution. The data are all in a standard data format using a dynamic template (Figure 2) such that they can be readily analyzed and/or viewed by staff members. The data recorded follow the SOAP acronym: Subjective, Objective, Assessment, and Plan. The data from each new patient can be compared to others like him or her at any time during this patient's journey through surgery or treatment. There are now structured phrases developed by JSCP for 307 outcomes (BOMs) and 1680 assessment items. Figure 3 shows the basic structure of a BOM per the JSCP. Because such templates are used to enter information for every patient, sufficient data are available to generate findings that can inform learning within the SKH. Comparisons can be made across numerous with similar or varying characteristics.

#### 2.2.2 Knowledge to performance/Practice (K2P)

The data from each patient, who comes to SKH, have been collected electronically, using the Basic Outcome Measure (BOM)‐based templates, since 2011. A tool was developed within the electronic medical record system to provide analytic views of the data for physicians and other medical staff members. The electronic medical record vendor is NEC, however, the visualization tool is called by SKH the “Novel Electronic Clinical Pathway Viewer” (NECV). In order to collect data efficiently, it is important to develop an information infrastructure based on standard structured data collection and review capabilities using tools such as NECV. With this tool, the data are “translated” into knowledge that can be leveraged to determine how best to proceed in terms of patient treatment by allowing the clinicians to observe how similar patients have responded, and to identify best practices overall. In other words, practice can be based on knowledge. Not only clinicians can access these data, but also everyone on the hospital team, including nurses, nutritionists, and others.

#### 2.2.3 Continuous learning and health improvement/Performance to data (P2D)

Since 1997, SKH has been conducting regular variance analyses to improve the healthcare process and clinical outcomes of its patients. In addition to the staff involved in treatment (eg, doctors, nurses, pharmacists, and physiotherapists), medical administrative staff, although not directly involved in treatment, are involved in certain aspects, such as cost analyses. Such multidisciplinary teamwork facilitates understanding of the disease process and provides opportunities for interprofessional communication with respect to the value of the care provided at this hospital for all patients.

The visualization tool, described in the prior section, provides an avenue for staff to be able to compare each patient to predecessors. These comparisons include care processes, assessments, costs, and outcomes, showing graphs and variances. Not only does this tool support learning, but more recently new methodologies such as “machine learning” have been applied to the data within SKH. Examples of the benefits of using such tools are provided in the following section. Lessons learned are applied to improve the health of new patients who enter this hospital, and the results are shared across the hospital and with interested healthcare providers in Japan and other countries.

“Clinical Pathways” Conferences provide another avenue to support continuous learning. These meetings have been held at SKH every other month for the past 20 years. At each meeting, one department within the hospital is responsible for analyzing information relevant to their department, and their patients, and presenting the results to all hospital staff, along with others from around the country and/or oversees. These are open meetings, and they typically have numerous visitors from other hospitals. The presenting team will consist of departmental staff with several different roles, including physician, anesthesiologist, nurse, nutritionist, administrator, systems engineer, and others. Recommendations for improving the care protocol/plan or Clinical Pathway(s) under discussion are made and discussed among the attendees. Results are thus disseminated to other hospitals. In addition, they are frequently shared through medical journals and scientific meetings. These recommendations, based upon results of research conducted on data from numerous patients, have resulted in modifications of the relevant Clinical Pathways within SKH to improve patient care, thus supporting continuous learning and a learning health system (LHS) within SKH. This process of continuous learning is referred to as *kaizen* in Japan. Examples are provided in the following section.

#### 2.2.4 Shared infrastructure

Hospitals within and outside of Japan are welcome to send representatives to the regular Clinical Pathways Conferences where care protocols and consolidated, long‐term patient data are presented and discussed. Thus far, there have been more than 1000 presenters, more than 24 000 inside participants in the path conferences over 20 years, and more than 6000 outside participants, and we continue to disseminate findings, not only in this region but also nationwide.

In addition to developing infrastructure, technology, and policies to support the internal LHS, SKH received a grant from Japan's AMED, which is akin to the NIH of the United States, to expand this concept to additional hospitals within Japan. In the ePath Project (AMED supported), where the author is the principal investigator, a data model was created by linking data of other tests and drugs and event information, such as surgery date or prescription date, which have a different format for each vendor. The converted data are stored in a repository of common specifications; after anonymization, integrated analysis across facilities and vendors is possible. (See https://e‐path.jp/index.html).

The ePath project (supported by AMED) is reinforcing the importance of consensus‐based standards for the collection of data leading to quality measurements. One of the initial steps in sharing and expanding this infrastructure was to convene meetings among representatives from the interested hospitals to review the BOM templates and definitions such that consensus could be built around implementing, adopting, and disseminating these standards, which are critical to being able to aggregate and analyze the data collected from patients at each of the participating hospitals.

#### 2.2.5 Culture

SKH has received and maintained the Gold Seal of Approval from the JCI since 2013.^10^ All staff are extremely proud of this accomplishment. The electronic approach to collecting data from each patient in standard formats/templates and aggregating large volumes of data that can enable clinicians to readily make informed decisions about the patient s/he is treating are at the heart of the hospital's quality initiatives. These processes and data collection and sharing are embraced by the various team members of each department and care team, and ingrained in the culture of this hospital.

Implementation of learning health system processes within SKH has proven useful in reviewing the medical process and efficiency and financial aspects of medical care. Revision of the clinical pathway or care protocol serves as an engine of the plan‐do‐check‐act (PDCA) cycle that is continuously operating to achieve best practices. Of course, such analysis poses a burden on frontline staff, so the hospital appointed a specific nurse to be responsible for instruction, dissemination, and analysis of the process, in addition to planning of the regular Clinical Pathway Conferences. Furthermore, with the availability of a large volume of information in electronic medical records, the SKH established a system whereby the Medical Information Department helps the medical team collect and analyze data upon request, thus taking the burden off the care team. The path revision initially required a period of 1 to 2 years, due to the resources requirements to analyze an adequate number of cases when collecting data on paper; however, the current process is much more rapid and can be done electronically when more than 100 cases are accumulated through our information system.

**FIGURE 4:**
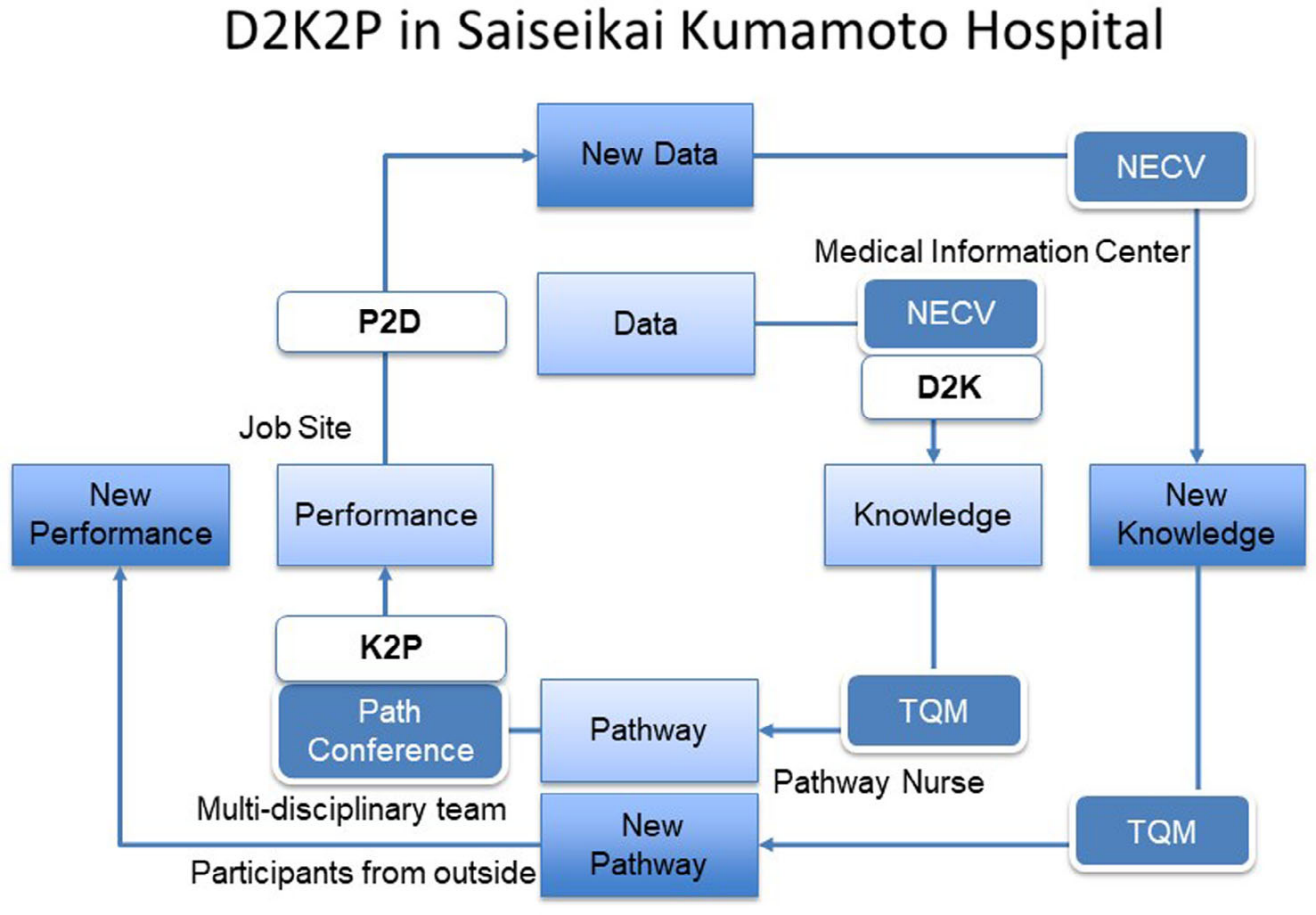
On the electronic pathway most medical data are automatically collected into DWH: variance data, lab test data, prescription, cost, DPC, etc. NECV can visualize path data and show medicine or lab data on a spreadsheet. TQM analyzes such real‐world data deeply and discusses with a Pathway Nurse. Finally, the new pathway is proposed and discussed further with participants from inside and outside during the Path Conference. Practice to Data (P2D), Data to Knowledge (D2K), and Knowledge to Practice (K2P) steps to support a learning health cycle are noted in this figure

The Total Quality Management group at the hospital is involved in ensuring that the knowledge is used to continuously improve upon processes across all hospital departments. The care team members at this hospital can all use this system and the related tools to assist them in making medical decisions for their patients, thus accelerating learning health cycles. The results from the regular Clinical Pathways are used to improve the care protocol or process, thus ensuring identification of best practices, and continuous improvement, and upholding the culture of *kaizen* (Figure 4). Note that Practice to Data (P2D), D2K, and K2P steps to support a learning health cycle are supported by this process.

### 2.3 Results: examples from the Saiseikai Kumamoto Learning Health System

**FIGURE 5:**
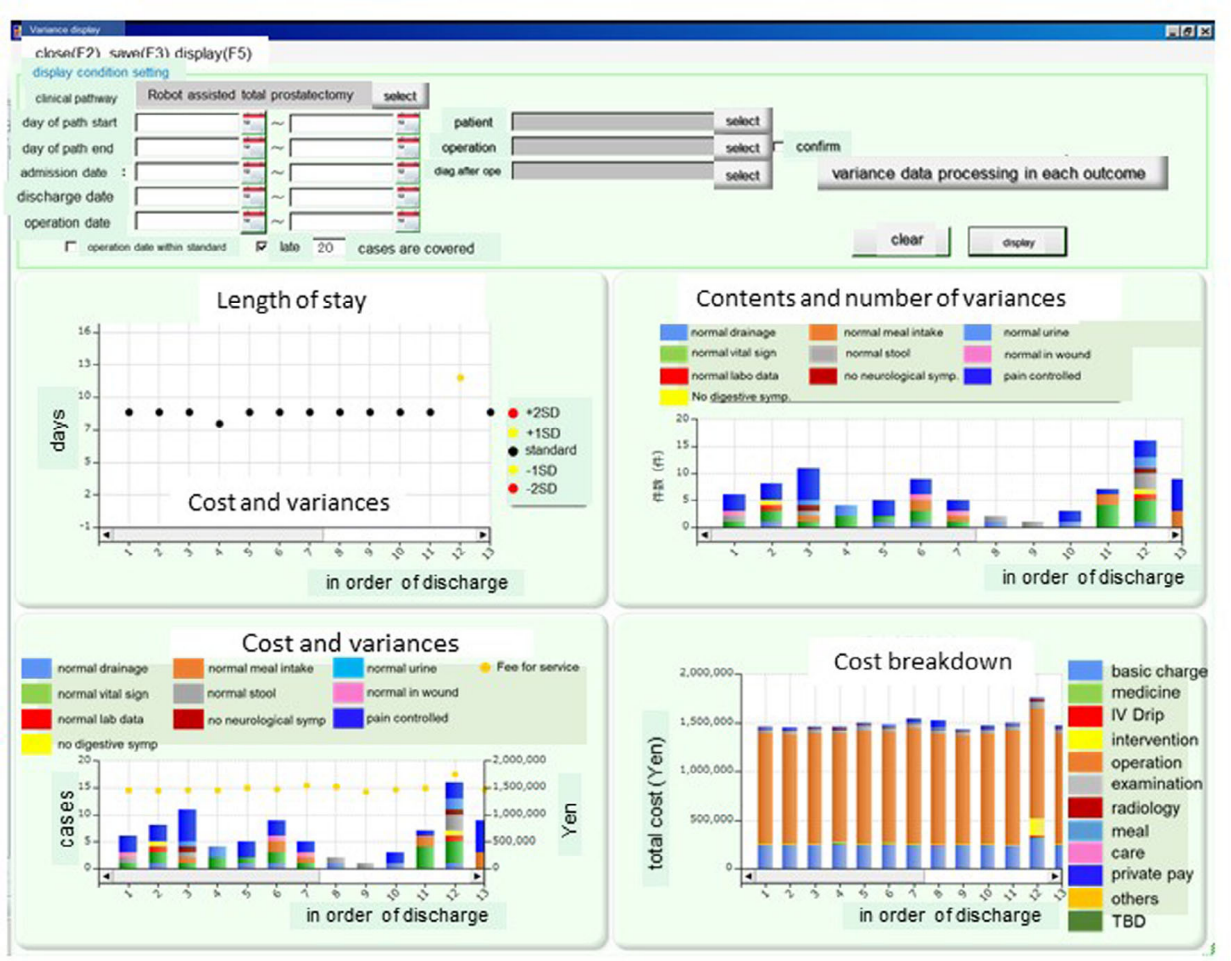
The main page of the Visualization Tool within the electronic medical record (NECV): All forms of variance data, including length of hospital stay and cost, are available via the variance display screens at all computer terminals in the Saiseikai Kumamoto hospital (SKH) for all staff to view. Every staff member can understand the medical process and patients who may be difficult to treat and types of extra costs that occur due to variances. Management staff can use such information to propose revisions to clinical pathways

In order to protect personal information, we obtain a signed consent form for data use at the first visit. In addition, research plans go through the in‐house ethics review committee. Different types of patient information, available at all times to SKH staff, are shown in Figure 5. This figure shows a view of the home screen (main page) of the visualization tool (NECV) within the EHR. There are four diagrams that are based upon measured and recorded assessments, and the variances from what is considered to be the norm: length of hospital stay (top left), cost (in Yen) and number of variances (bottom left), type of assessment and number of variances by day of hospital stay (top right), and cost breakdown (bottom right).

#### 2.3.1 Establishing a Prevention Program for Health Improvement based on K2P

**FIGURE 6:**
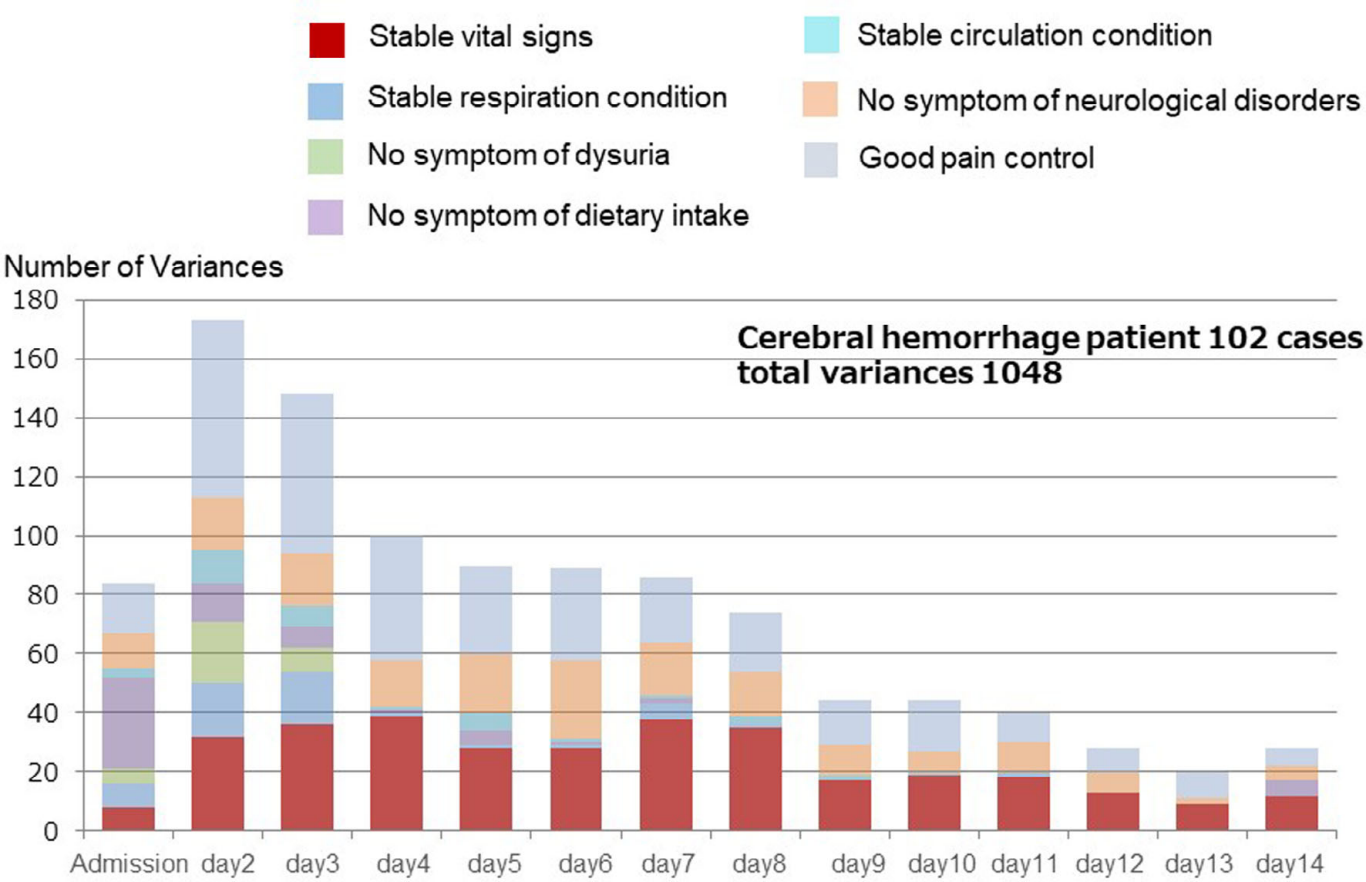
Variance analysis in 102 cerebral hemorrhage patients over time: The outcomes related to assessment item can be easily extracted electronically from the clinical pathway of brain hemorrhage. Forty‐eight patients of 102 cerebral hemorrhage patients presented with fever caused by infection, of which 25 patients had aspiration pneumonia. Preventing aspiration pneumonia is clearly critical. Thus, the revision of the pathway was started by the multidisciplinary team

**FIGURE 7:**
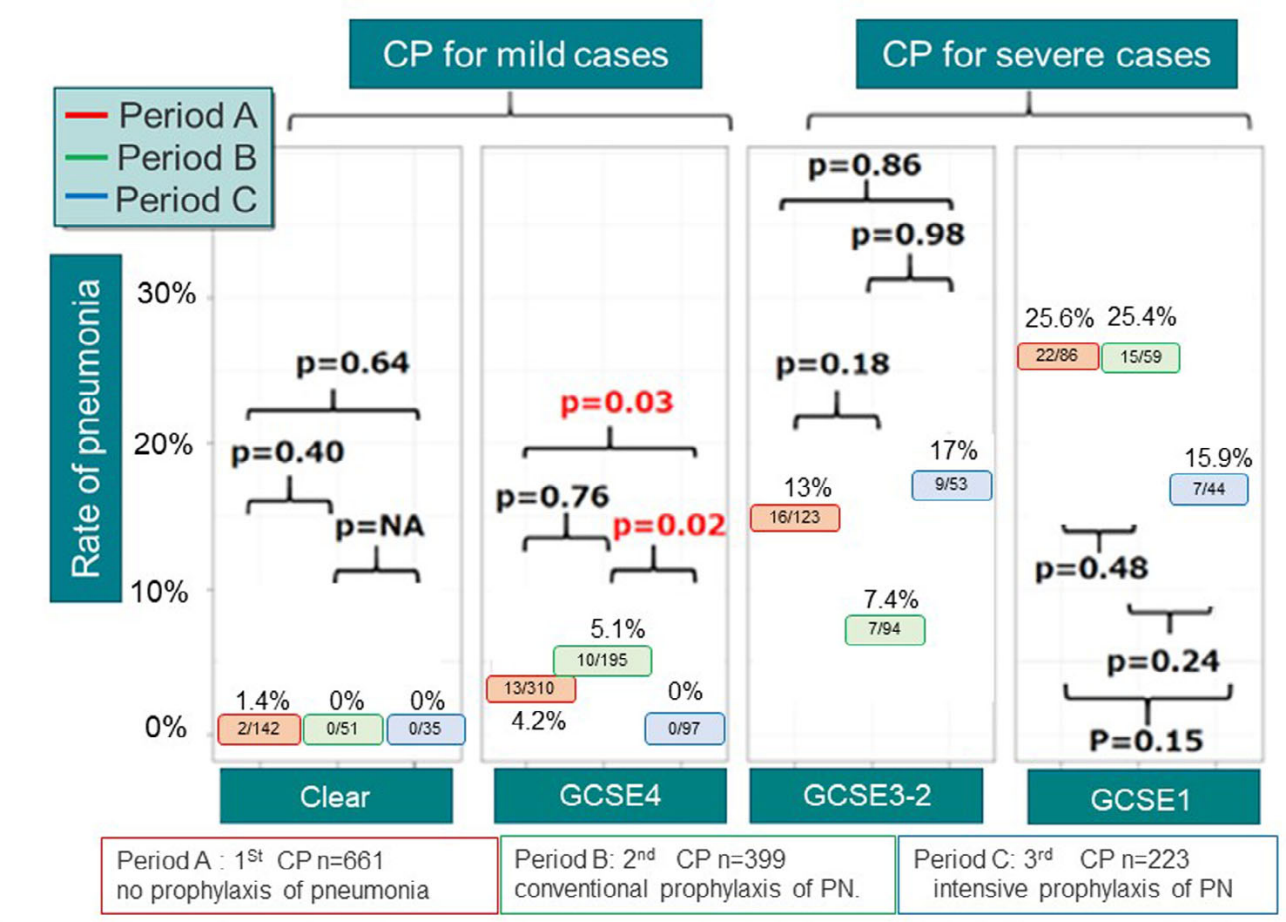
Three pathways have been used over three periods: A (no prophylaxis for pneumonia), B (conventional prophylaxis), and C (intensive prophylaxis). The integrated oral hygiene has been included in the clinical pathway during Period C. The rate of pneumonia has been decreased, especially in mild cases of stroke. The average age was 71.3 ± 13.6 years. GCSE; Glasgow Coma Scale eye opening JCSI≒ GCSE4 JCSII≒ GCSE3‐2 JCSIII≒ GCSE1

An example of knowledge‐based practice that led to the establishment of a prevention program is depicted through Figure 6. Figure 6 shows the variance tracking by day of hospital stay for 102 patients with cerebral hemorrhage (brain stroke). The total number of variances recorded was 1048. Their distribution by day is depicted by each of the columns in this figure; as variances decrease, discharge from the hospital becomes more viable. Of the 102 patients in this graph, 48 patients had a total of 332 variances that were found to be related to “unstable vital signs,” and 189 of these variances were specifically related to fever (temperatures measuring over 37.5° Centigrade). These 48 patients were explored further and were found to have infections. Twenty‐five of the infections were due to aspiration pneumonia, whereas the other 23 had urinary tract or other sources/types of infection. The hospital staff recommended a change to the routine care protocol (Clinical Pathway) to focus on establishing a prevention program for aspiration pneumonia. New processes included additional assessment items for early detection of pneumonia, implementing a test plan for early diagnosis, maintaining good oral hygiene, and the use of a 30° bed angle for recovering patients. Figure 7 shows the rate of pneumonia during 3 periods during which different Clinical Pathways (care protocols) for brain stroke patients were followed. The average age was 71.3 ± 13.6 years. With respect to type of stroke, 30% was subarachnoid hemorrhage and 70% was intracerebral. As shown in Figure 7, knowledge‐based changes to the Clinical Pathway (period C) resulted in a significant decrease in aspiration pneumonia for patients with stroke, especially when the “intensive prophylaxis” protocol was used in mild cases (*P* value .02).

These learnings and suggestions for improvements were discussed, based on the variance collection and analysis results, thus leading to a revision of the care protocol/Clinical Pathway and completion of a cycle of quality improvement. This is an example of the results obtained through organizational and sustained efforts through which clinicians performed variance analysis, using the data collection and visualization system. The suggested knowledge‐based improvements then incorporated them into a revised Clinical Pathway, which exemplifies K2P. It is important to systematically improve the learning health cycle for continuous improvement. This specific example and the revision process have been reported in detail by Matsumoto et al.^13^

#### 2.3.2 Machine learning methodology to support K2P and continuous learning

**FIGURE 8:**
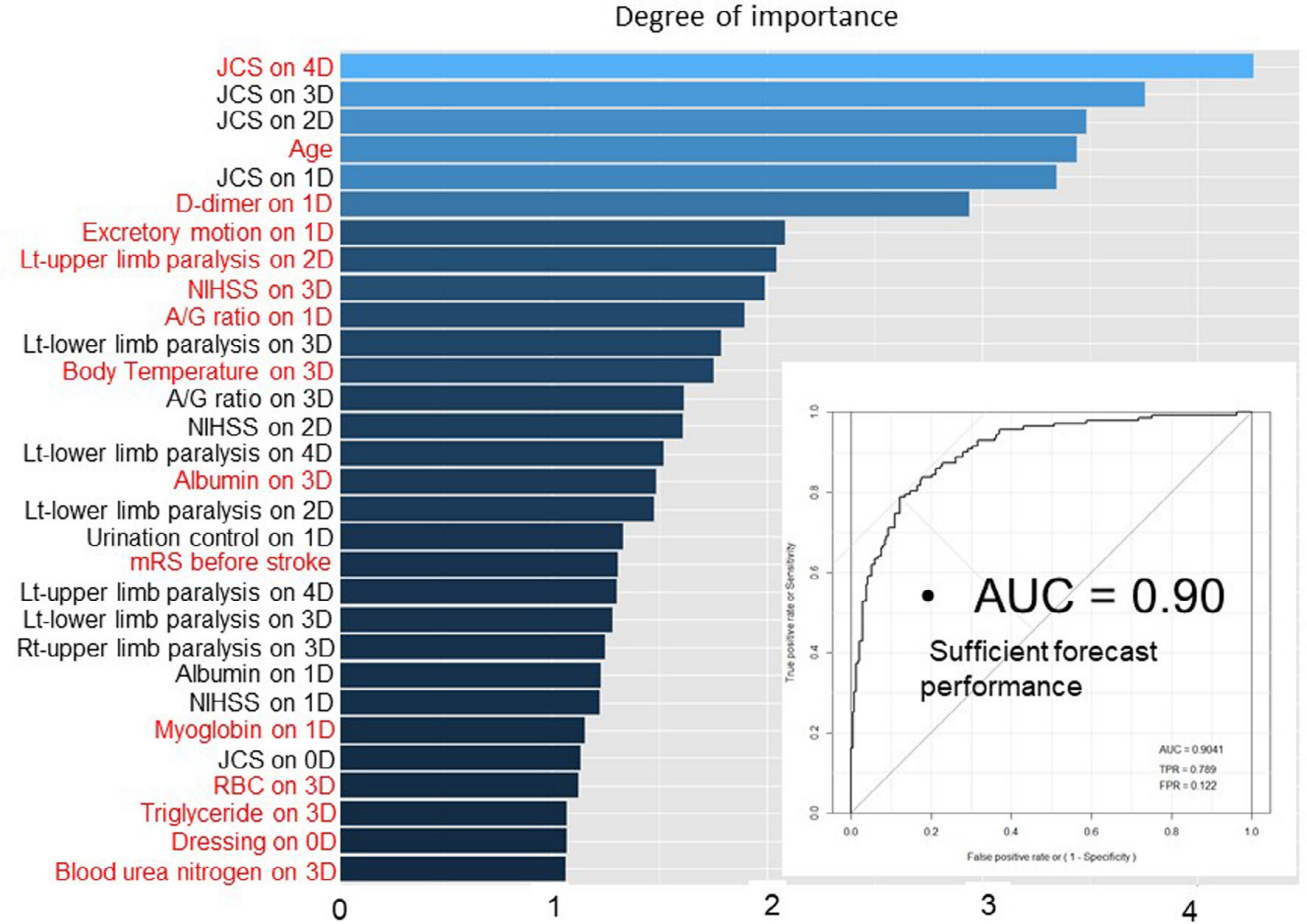
Machine learning—random forest analysis of data from 379 cerebral infarction patients. The target variable is discharged on the 8th day. Top 30 of 1835 variance items on each hospital day were showed. 0D means at the emergency. Red indicates first appearance. Null is converted to Median. JCS (Japan Coma Scale); NIHSS (National Institute of Health Stroke Scale); mRS (modified Rankin Scale)

The use of machine learning algorithms was applied to patient data for 379 patients suffering from cerebral infarction. There were 1835 variance items (assessments that varied from what was considered a normal range) included in this analysis. Figure 8 shows the results of a random forest machine learning algorithm that exposed unexpected factors, which could influence future therapy, for certain, of these patients. In a collaborative study between SKH and Kyushu University, the learning algorithm used “discharge on the 8^th^ day” as an objective. The top factors that influenced this discharge objective (from strongest to weakest) were Japanese Coma Scale score, age, D‐dimer level, albumin/globulin ratio, and albumin level. An AUC of 0.90 indicates a relatively high explanatory power. In this random forest analysis, the handling of null is done by inserting the median for the calculation. Further studies must be done; however, this could indicate that there is an unanticipated relationship between the albumin/globulin ration that relates to discharge day.

#### 2.3.3 Regular Clinical Pathways Conferences as a Culture of Learning

The 122nd Clinical Pathways (CP) Conference was held on 5 December 2018. These conferences take place at SKH every 2 months and are open to all interested parties from the hospital, Japan, and the world. This is one aspect of a learning health system culture that has been ingrained within this hospital for more than two decades. At the 122nd conference, which is used as an example, the surgery department was responsible for analyzing data related to their procedures/care protocols and presenting their findings to the attendees. Every 2 months, a different department has this responsibility. The surgery department is somewhat unique since they service many other departments within the hospital.

**FIGURE 9:**
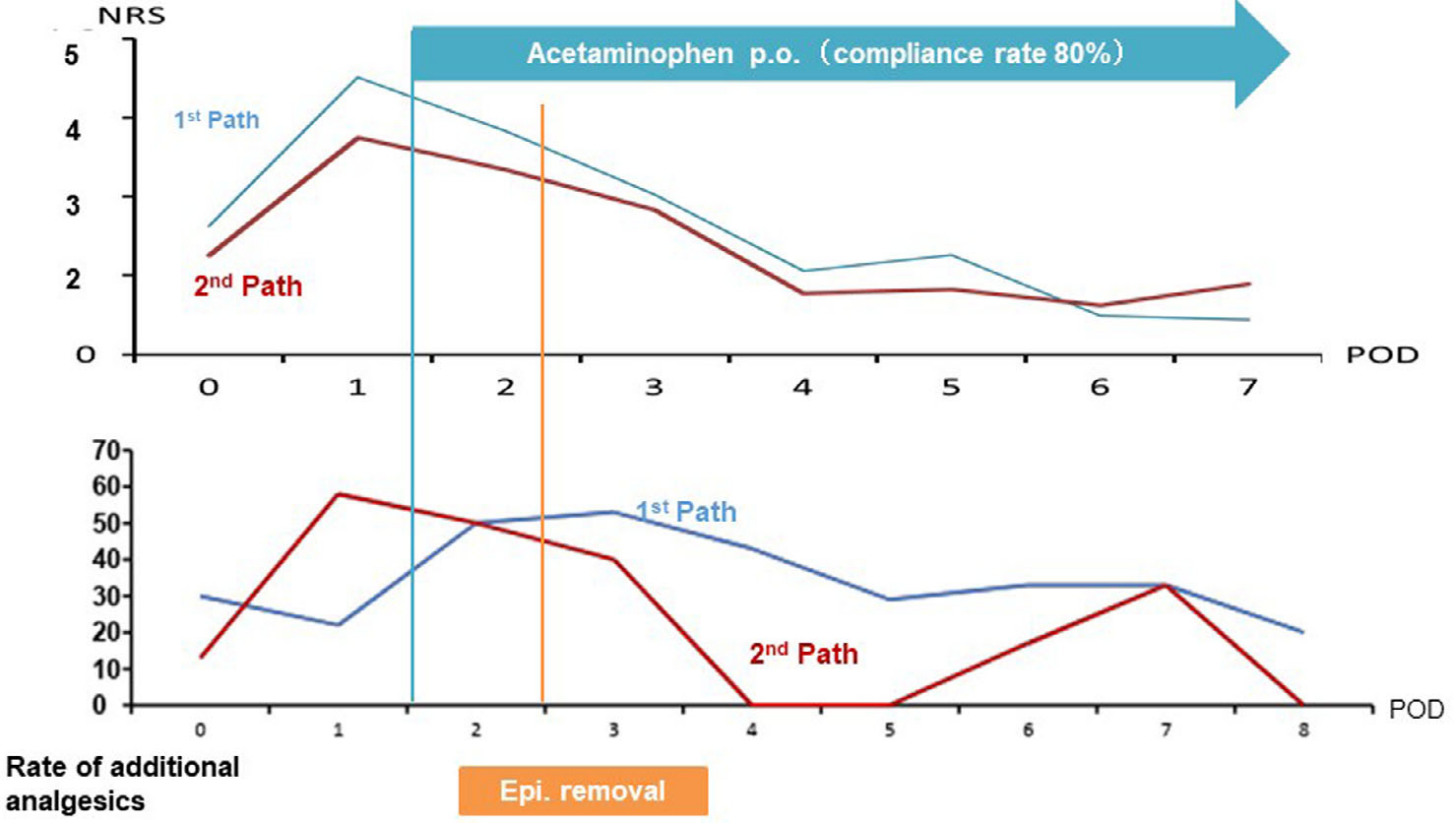
The first pathway was used from 2014.5 to 2015.9 in 112 cases of laparoscopic colectomy and the second one from 2015.10 to 2016.3 in 67 cases. After pathway revision, acetaminophen sufficiently reduced pain and the use of additional analgesics was reduced. Moreover, the median hospital stay decreased from 10 to 8 days. NRS (Numerical Rating Scale of pain)

Those presenting results of Clinical Pathways data analyses at this 122nd CP conference included an anesthesiologist, a doctor, two nurses, a clinical engineer, and an administrator. Their topic of focus was laparoscopic surgery and their goal was enhanced recovery after surgery (ERAS) that would shorten the length of stay in the hospital. The initial Clinical Pathway (care protocol) was developed in 2011. At this meeting, the team looked at anesthesia methods, use of catheters, impact on the EMR system, and surgery scheduling. Figure 9 shows the effect of acetaminophen on reducing pain and the use of additional analgesics for two different Clinical Pathways/care protocols, which were implemented from May 2014 through September 2015 and between October 2015 and March 2016. Analysis of the variances from acceptable pain control in laparoscopic colectomy showed that pain control was not sufficient in 112 cases in the first stage. However, changing the method of pain control through the use of acetaminophen in the second stage indicates that the NRS (Numerical Rating Scale) score was decreased compared with the first stage, thus decreasing the rate at which additional analgesics were needed.

Another recommendation related to anesthesia during this Conference was, for patients receiving nerve blocks in a smaller area (vs an epidural), the use of a catheter may not be necessary; this, in turn, translates to the patient spending less time in the hospital post‐surgery. Patients undergoing a cholecystectomy were analyzed; there were 148 cases included. The most influential factors on the operation time were inflammation (cholecystitis) and BMI (Body Mass Index). At the conference, the criteria for urethral catheter were discussed and it was suggested that a catheter is not necessary for the cases without inflammation and BMI under 24, which make up about 78% of cholecystectomy cases. See Table 1. There is also the option for these patients to control pain locally vs whole body pain control (eg, by opiates).

**TABLE 1:** Total of 148 cases of laparoscopic cholecystectomy was investigated from Jan to Sep. in 2018

	Inflammation	No inflammation
BMI 24 >	Depend on patient	No catheter
BMI 24 ≤	Catheter	Depend on patient

*Note*: The factors with the greatest influence on operation time over 2 h were inflammation and BMI (odds ratio: 5.0, 3.0; *P* value: .01, .03). Urethral catheters have been unnecessary in 78% of patients. Criteria for indication of urethral catheter during laparoscopic cholecystectomy.

The systems engineer at this Clinical Pathways Conference commented that every personnel role has a customized view of the data and that, for the surgery department, the systems engineers try to ensure three opportunities: (a) precise data, (b) matching surgery data with cost data; and (c) managing the master templates to obtain these data. The hospital administrator presented on the importance of cooperation in terms of the surgery and when a catheter is used. This individual also commented that scheduling the surgery should be based upon BMI, specifically that patients with a BMI over 24 typically require a longer period of time for the surgery, and that it is important to maintain quality along with efficiency.

The findings of the surgery team were discussed with questions and comments from the audience. This team will update the Clinical Pathway as deemed appropriate based upon the knowledge gained (K2P).

## 3 DISCUSSION

SKH has worked diligently for over two decades toward developing “Clinical Pathways” that are essentially learning health cycles within the hospital. This hospital also exudes a culture of quality and continuous learning, collecting data from each patient in a common template such that it can be readily aggregated and compared with similar data from previous patients with the same diagnosis or problem. In addition, the information infrastructure has played a key role in encouraging compliance and making this growing body of knowledge through readily comprehensible and accessible views into the data and analyses for all hospital staff. The learnings are readily shared, discussed, and disseminated.

Although it is acknowledged that there is more work to be done, the SKH processes, infrastructure, and culture appear to be similar, or heading in a common direction, when compared with the published attributes of a fully functional LHS. This institution has achieved the basic process of a learning health cycle (D2K, K2P, and P2D) along with an information infrastructure that makes the knowledge readily available for all staff and a culture that proudly supports and adheres to collecting structured data from each patient and comparing this to others like that patient. The dissemination of information and continuous learning is also supported by this hospital and ingrained in the culture where the staff believe in providing the best care for each patient at a lower cost to each patient.

The leaders at SKH have now begun to work with other hospitals to expand this LHS. Although there was an effort made to use common definitions for outcome measures, as developed through consensus‐based processes within the JSCP, not all hospitals in Japan, or other countries, have used the same outcome measure definitions. Expanding the Kumamoto experience involves building consensus among these other hospitals around the outcome measures and assessments that are used for evaluating and reporting a “variance” from the desired outcome.

The development and adoption of standards are challenging, especially when vendors of EHR systems and research networks introduce their own proprietary models and standards. This is the crux of the challenge in achieving true interoperability, especially semantic interoperability, which includes the exchange of data along with the meaning of that data. Interoperability is but one of the barriers that must be addressed before achievement of a global learning health system is actually on the horizon. Defining the basic measures for desired outcomes represents significant progress in terms of creating consensus‐based standards for collecting data that can support the generation of knowledge for an LHS. Leveraging the experience gained by SKH staff from large volumes of data in a common, analyzable electronic format demonstrates that this is possible on an institutional scale. A willingness to learn from this information and disseminate it broadly is a testimony to a culture that will encourage others that LHSs are indeed possible.

In addition to LHS continuous improvement activities and *kaizen*, the data gathered as an integral aspect of routine patient care will be useful in innovative areas in the medical process. For example, research studies can be conducted by comparing a clinical pathway for an investigational agent with a similar pathway involving identical basic medical procedures, tests, and treatments with placebo in a multicenter study. Such “real world data” used for research purposes may, in turn, reduce the cost of new drug development, and thus has social significance. A database of EHR information in standard formats can also be readily be compiled, producing a valuable resource for large‐scale clinical studies, new drug discovery, and post‐market data collection regarding adverse events (safety surveillance). Furthermore, such data can be used for optimum planning (eg, therapy, test, and dosage regimen) and designs of future research studies. Outcomes of these studies may lead to development of programs that assist diagnosis^14^, ^15^ and medical examination, and, ultimately, to development and implementation of extended machine learning and AI applications.^16^, ^17^, ^18^, ^19^ Clinical data stored in many electronic medical records systems are still not fully used because data input and retrieval systems are not well defined.

The SKH experience and its information infrastructure provide a functional LHS that can provide a representative sample for an LHS Maturity Model. The experience from this LHS is now being shared more broadly with the hope that it can be scaled and serve as a solid platform for measures aimed at improving medical care, thus establishing broader learning health systems (LHSs).

## CONFLICT OF INTEREST

All authors declare that they have no conflicts of interest.
